# Supplementary figures and images for: Synergies between urban heat island and heat waves in Seoul: The role of wind speed and land use characteristics
Source: PLoS One. 2020 Dec 7;15(12):e0243571. doi: 10.1371/journal.pone.0243571 (PMC7721160; doi:10.1371/journal.pone.0243571)

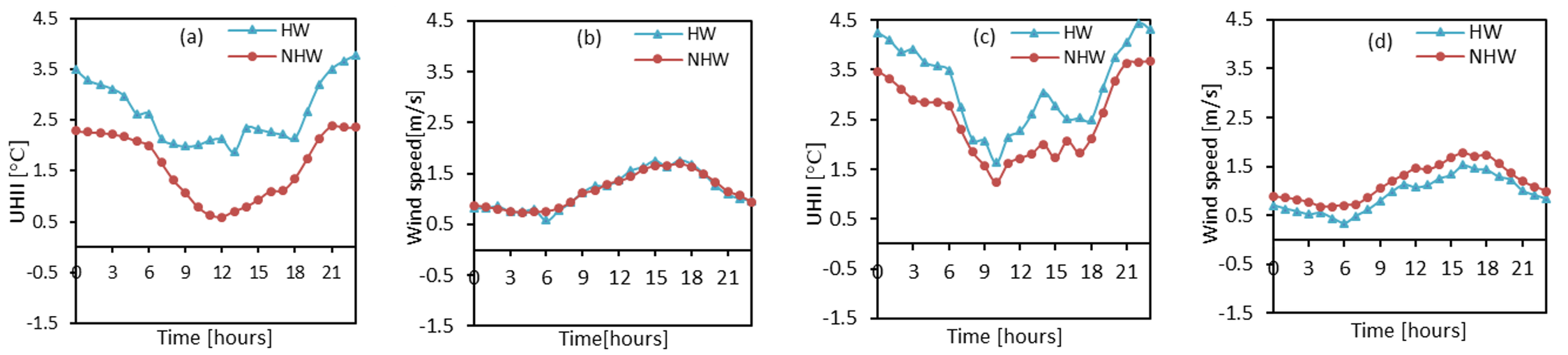

Supplement: S1 Fig — (a) UHII_2012 (b) wind speed_2012 (c) UHII_2016 (d) wind speed_2016. (TIF) [file pone.0243571.s001.tif]

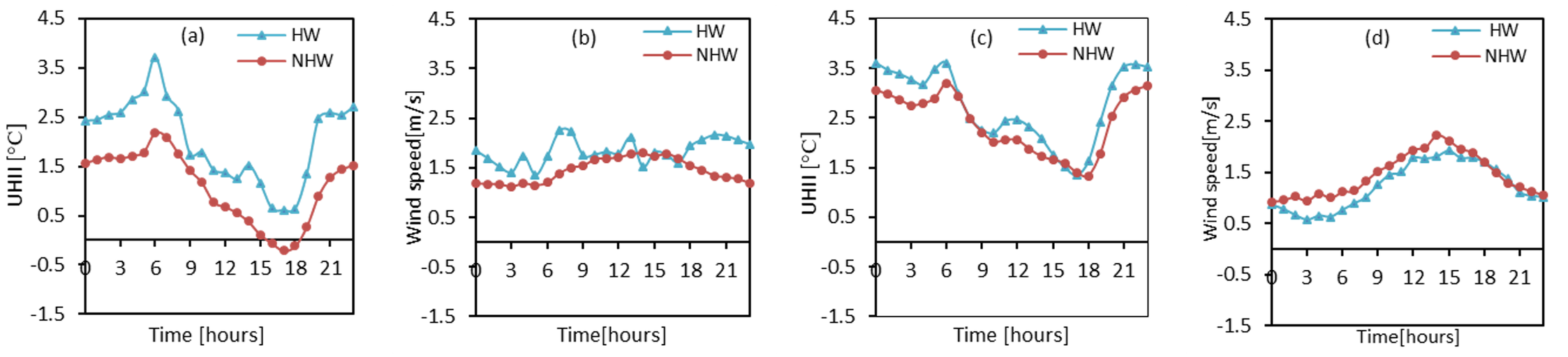

Supplement: S2 Fig — (a) UHII_2012 (b) wind speed_2012 (c) UHII_2016 (d) wind speed_2016. (TIF) [file pone.0243571.s002.tif]

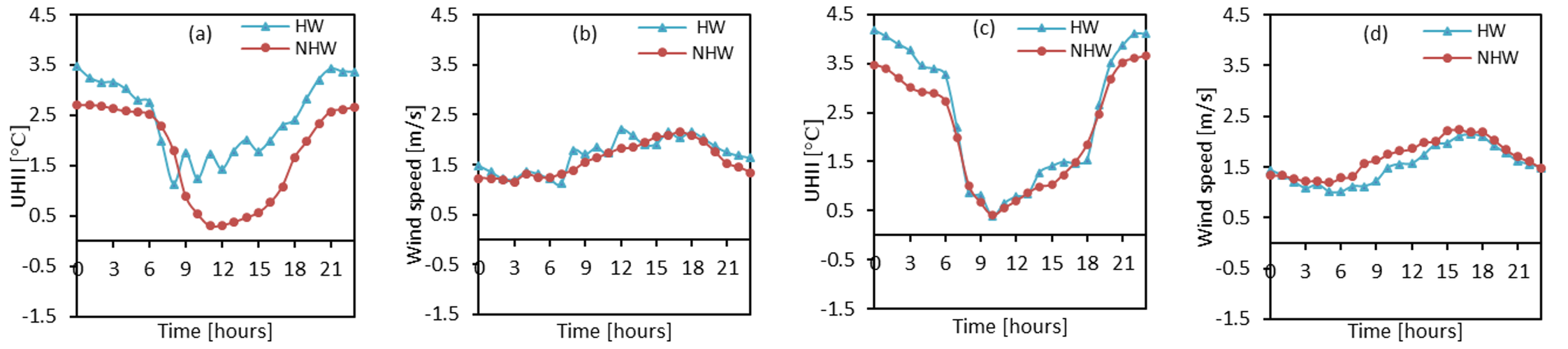

Supplement: S3 Fig — (a) UHII_2012 (b) wind speed_2012 (c) UHII_2016 (d) wind speed_2016. (TIF) [file pone.0243571.s003.tif]

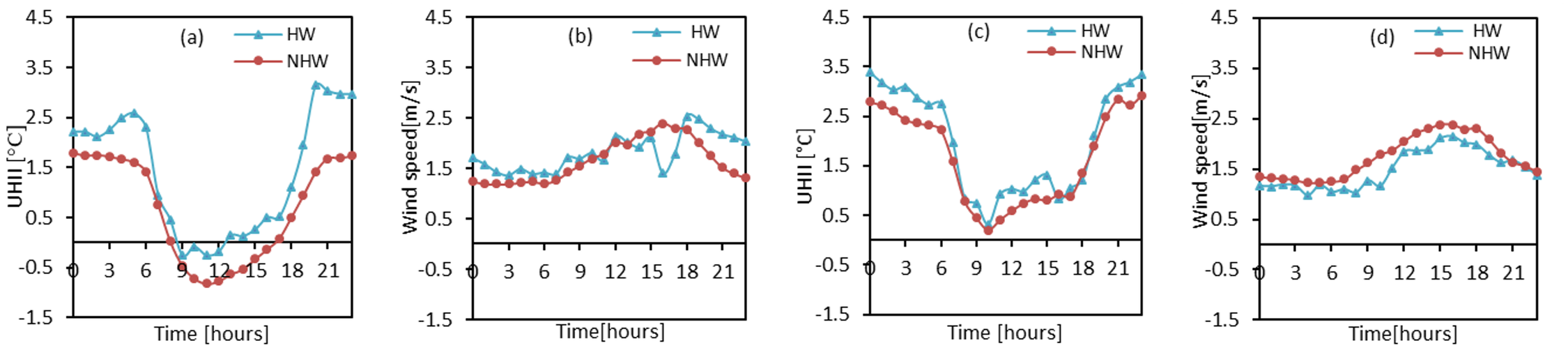

Supplement: S4 Fig — (a) UHII_2012 (b) wind speed_2012 (c) UHII_2016 (d) wind speed_2016. (TIF) [file pone.0243571.s004.tif]

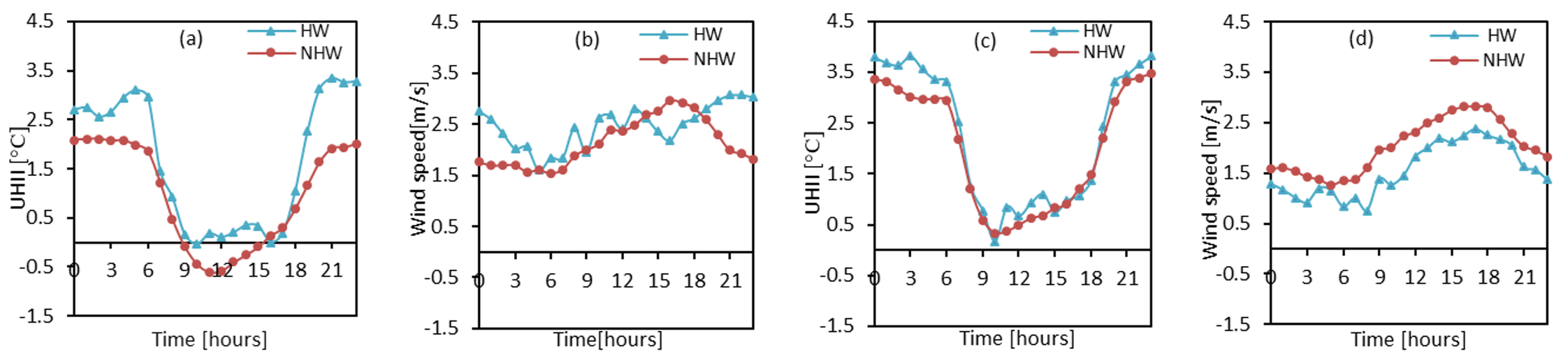

Supplement: S5 Fig — (a) UHII_2012 (b) wind speed_2012 (c) UHII_2016 (d) wind speed_2016. (TIF) [file pone.0243571.s005.tif]

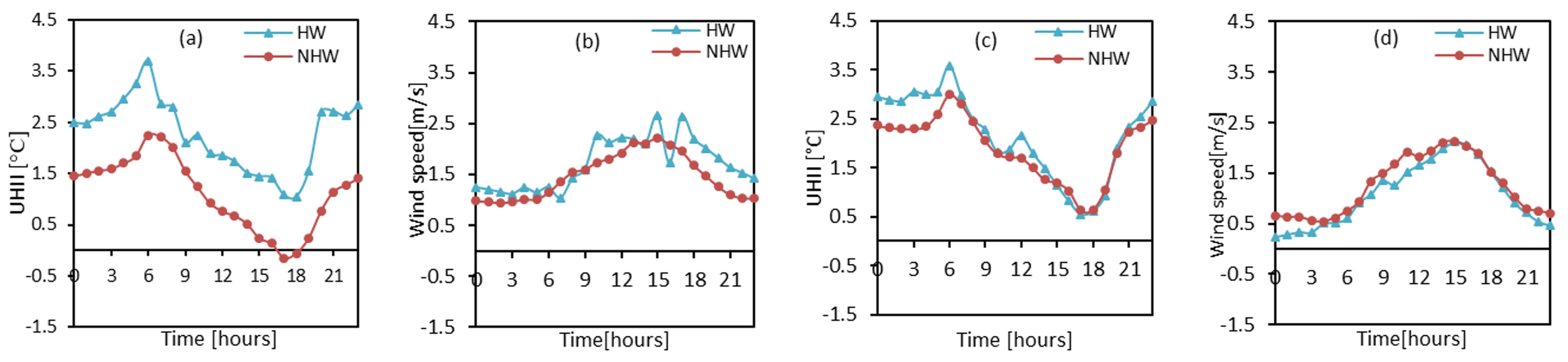

Supplement: S6 Fig — (a) UHII_2012 (b) wind speed_2012 (c) UHII_2016 (d) wind speed_2016. (TIF) [file pone.0243571.s006.tif]

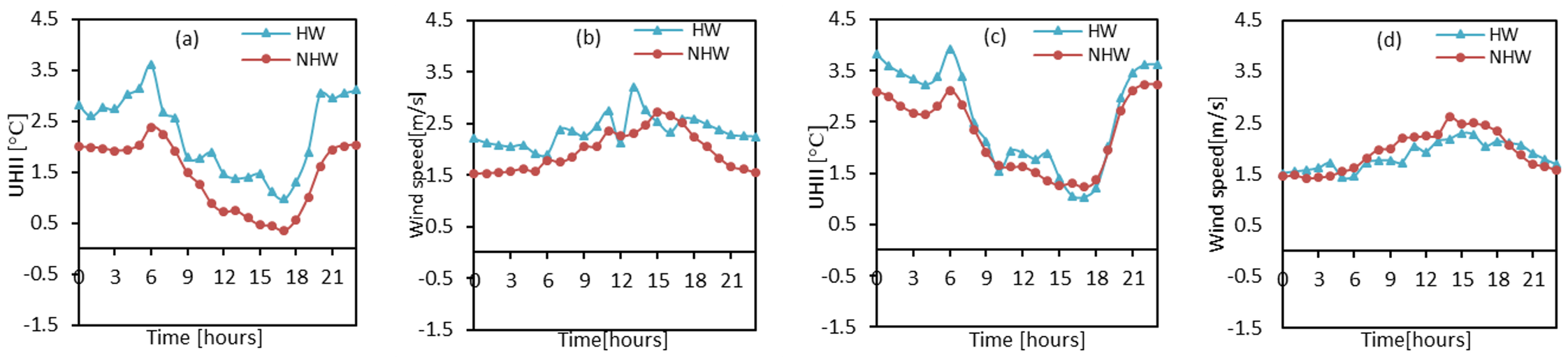

Supplement: S7 Fig — (a) UHII_2012 (b) wind speed_2012 (c) UHII_2016 (d) wind speed_2016. (TIF) [file pone.0243571.s007.tif]

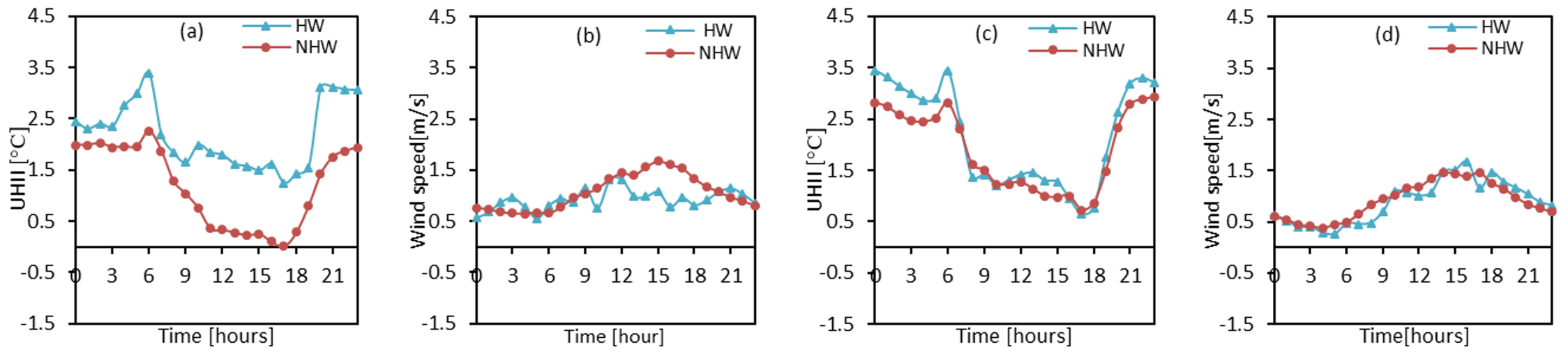

Supplement: S8 Fig — (a) UHII_2012 (b) wind speed_2012 (c) UHII_2016 (d) wind speed_2016. (TIF) [file pone.0243571.s008.tif]

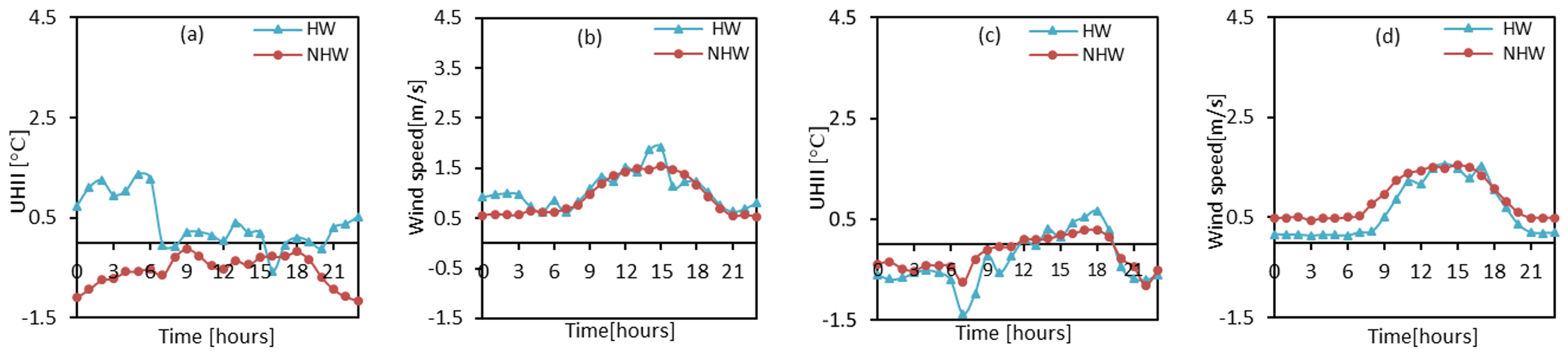

Supplement: S9 Fig — (a) UHII_2012 (b) wind speed_2012 (c) UHII_2016 (d) wind speed_2016. (TIF) [file pone.0243571.s009.tif]

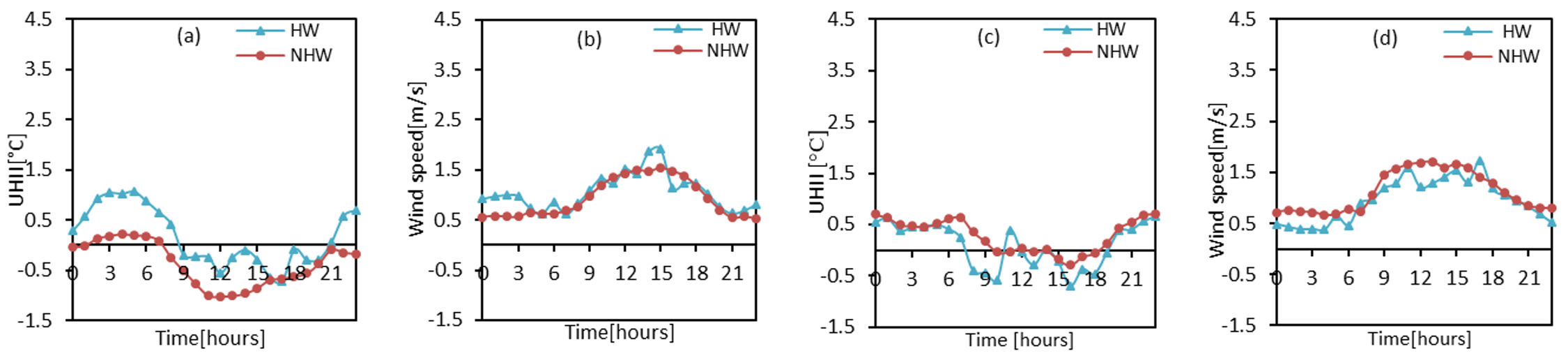

Supplement: S10 Fig — (a) UHII_2012 (b) wind speed_2012 (c) UHII_2016 (d) wind speed_2016. (TIF) [file pone.0243571.s010.tif]
